# Editorial: Recent advances in attempts to improve medication adherence-from basic research to clinical practice

**DOI:** 10.3389/fphar.2023.1144662

**Published:** 2023-02-08

**Authors:** Przemyslaw Kardas, Alexandra L. Dima, Ines Potočnjak, Bjorn Wettermark, Tamas Agh

**Affiliations:** ^1^ Medication Adherence Research Center, Department of Family Medicine, Medical University of Lodz, Lodz, Poland; ^2^ Health Technology Assessment in Primary Care and Mental Health (PRISMA) Research Group, Institut de Recerca Sant Joan de Déu, Barcelona, Spain; ^3^ Department of Social Psychology and Quantitative Psychology, University of Barcelona, Barcelona, Spain; ^4^ Institute for Clinical Medical Research and Education, University Hospital Center Sisters of Charity, Zagreb, Croatia; ^5^ Department of Pharmacy, Faculty of Pharmacy, Uppsala University, Uppsala, Sweden; ^6^ Faculty of Medicine, Vilnius University, Vilnius, Lithuania; ^7^ Syreon Research Institute, Budapest, Hungary; ^8^ Center for Health Technology Assessment and Pharmacoeconomic Research, University of Pécs, Pécs, Hungary

**Keywords:** medication adherence, clinical practice, basic research, Europe, healtcare systems

Adequate implementation of evidence-based pharmacotherapies is an obvious precondition for their effectiveness in real-life settings. Indeed, ‘Drugs do not work in patients who do not take them’, as the well-known quote by C. Everett Koop, US Surgeon General, says (Everett Koop, 1985). Unfortunately, despite more than half a century of dedicated research, corrective and awareness-raising activities, medication adherence still remains far from perfect. Twenty years ago, the World Health Organization released its seminal report on adherence (World Health Organization, 2003), which popularised the memorable number of as many as 50% of patients deviating from their prescribed treatment. Even if it may be assumed that these statistics seriously simplify the problem of non-adherence, there are also good reasons to believe that this proportion was not overestimated. What is worse, current statistics of non-adherence are not much different (Foley et al., 2021).

**FIGURE 1:**
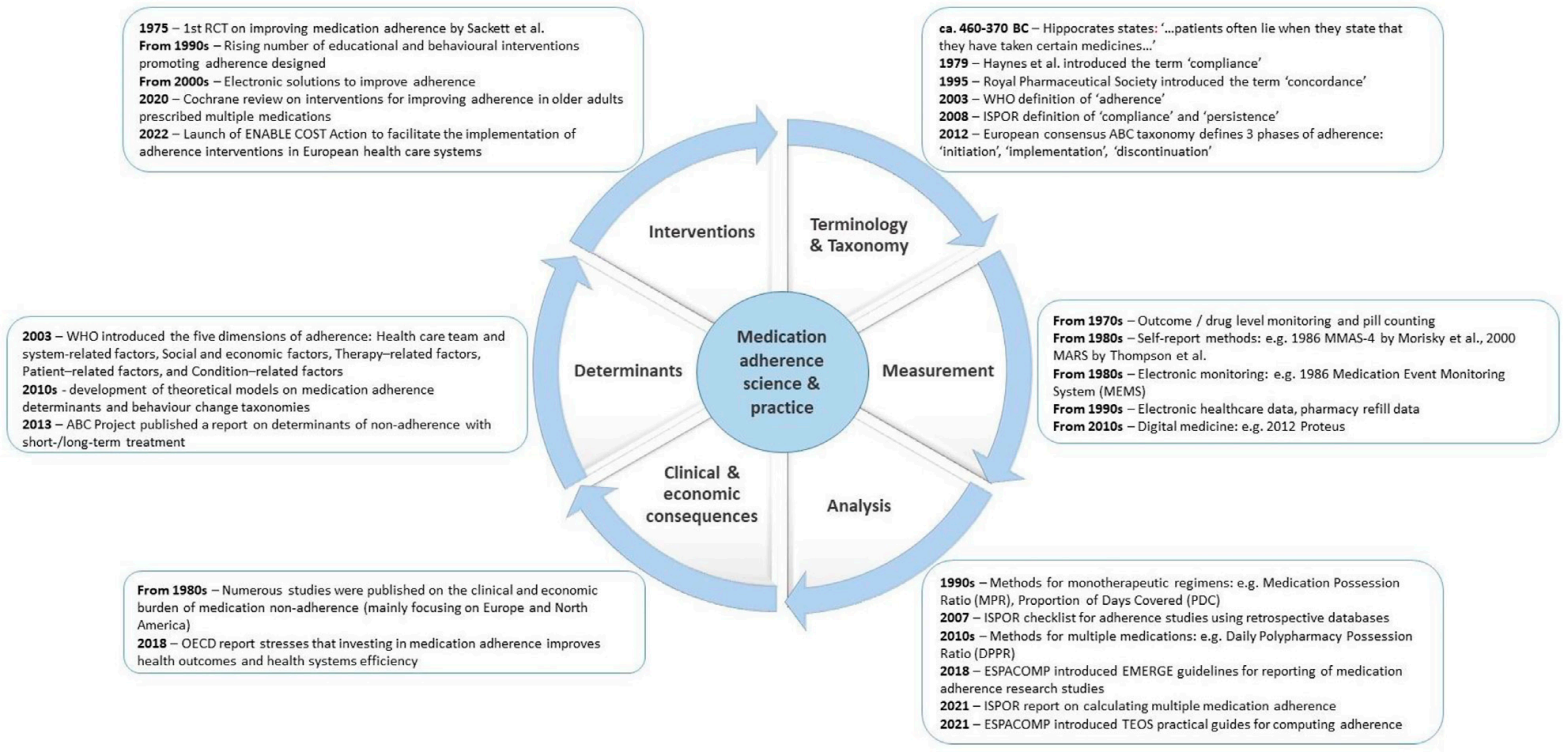
Milestones of medication adherence-related science and practice. Notes: ENABLE—European Network to Advance Best practices and technoLogy on medication adherencE” (ENABLE) COST Action, ESPACOMP—International Society for Medication Adherence, ISPOR - International Society for Pharmacoeconomics and Outcomes Research, MARS - Medication Adherence Report Scale, MMAS-4—4-Item Morisky Medication Adherence Scale, OECD—Organization for Economic Cooperation and Development, WHO—World Health Organisation.

An analysis of the milestones of medication adherence research and practice (Figure 1) proves that patients’ deviations from prescribed treatment are as old as the medicine itself. Hippocrates, the father of medicine, was the first one to make a note of what we now call non-adherence. This phenomenon has since not only caused frustration for thousands of practitioners, but has also been the object of interest for thousands of researchers. As a result, currently conducted searches of scientific literature databases using medication adherence terms return over 100,000 records. What can we learn from that bulk of publications?

One practical lesson is that a magic wand that would solve the puzzle of non-adherence simply does not exist. Taking medications as prescribed is a human behaviour driven by many interlinked factors. Therefore, a single one-size-fits-all solution is unlikely to be found. If so, should we abandon our hope to improve adherence? Some inspiration could be drawn from road traffic: no single intervention makes it 100% safe, yet several improvements (e.g., airbags, speed limits, *etc.*) proved to work, and their collective application has produced an additive effect in saving drivers and passengers’ lives.

Such a perspective created the background for this Research Topic of “Frontiers in Pharmacology”. When designing it, we aimed to cover the full spectrum of issues and solutions (Figure 1), which, when brought together, may help improve medication adherence. In response to this call, a wide range of modern approaches and innovative technologies has been described, from new survey instruments (Larsen et al.), to electronic pillboxes (Goetzinger et al.). Tackling adherence in real-life conditions, studies investigated new, unexpected factors affecting adherence: the COVID-19 pandemics (Malo et al.), and war hostilities (Khanyk et al.).

Unlike the other tools that mostly assess the level of adherence, OMAS-37 looked at the causes of non-adherence (Larsen et al.). Exploring various barriers to proper drug taking, it proved to be a valid and reliable instrument which may be a good starting point for further interventions. Another approach has been used by (Kostalova et al.), who measured tacrolimus concentration in kidney transplant recipients. Its intra-patient variability seemed to be an easy-to-use marker of non-adherence to this life-saving therapy. Finally, using tree-based prediction models (Wendl et al.), helped to identify target groups and individuals for adherence interventions in typical chronic conditions of diabetes type 1, type 2 and hyperlipidaemia. Notably, their approach also allowed to predict the economic consequences of interventions.

Two studies of our collection assessed adherence to statins. Interestingly, a Dutch study in diabetes type 2 patients found relatively high levels of adherence (Beernink et al.). Nevertheless, several easy to assess factors, such as higher HbA1c and higher BMI, correlated with lower adherence, and not attaining the LDLc level. Based on an analysis of longitudinal trends of statin use in new users before and during the COVID-19 pandemic, the following four patterns were identified: high adherence (37.2% of subjects); low adherence (35.6%); occasional use (14.9%); and gradual decline (12.3%) (Malo et al.). A study in Indonesia (Alfian et al.) found the level of self-reported non-adherence to antihypertensive treatment to be 41.8%. Among other factors, patients’ awareness of hypertension and emotional burden due to this condition correlated with non-adherence. These findings can form a solid basis for selecting patients in need of adherence support, and finding appropriate ways to support them, thus tailoring interventions to the relevant determinants.

Perhaps, the best interventions are those targeted at relevant determinants in a way that is acceptable to patients. As illustrated by (Barnestein-Fonseca et al.), individual training significantly improves inhalation technique in older adult COPD patients. However, novel technologies are also well-received: as many as two-thirds of breast cancer survivors declared that they would accept a medication adherence enhancing eHealth technology (electronic pillbox connected to a smartphone application) to improve daily adherence to their adjuvant endocrine therapy [Goetzinger et al.).

In the light of these findings, it is frustrating that European countries give medication adherence management low priority. A pan-European study identified 13 reimbursed medication adherence enhancing interventions (MAEIs) in nine countries only (Ágh et al.). The countries with a higher GDP *per capita* tend to have more reimbursed interventions. Is it because they can afford that? Or just the opposite: maybe due to better care for medication adherence these countries are wealthier? Some inspiration can be drawn from Ukraine: the country, under the unfavourable conditions of hostilities, tries to do its best to maintain long-term treatment of their citizens (Khanyk et al.), assuming that the human capital is crucial for its existence.

Looking forward, we have to accept the simple fact that medication non-adherence will remain a challenge. The aging of the global society, the rising tide of non-communicable chronic conditions, multimorbidity and associated polypharmacy, as well as new global challenges, such as the COVID-19 pandemic, are likely to create new barriers to medication-taking as prescribed (Kardas et al., 2021; Ágh et al., 2021). In the recent years, as illustrated by publications in this issue, medication non-adherence ceased to be merely a ‘patient problem’ and is now considered an important indicator of the quality of care within healthcare systems. Therefore, instead of being blamed, patients need to be supported in their therapeutic journeys. To enhance adherence, all stakeholders need to collectively create adherence-enabling environments. MAEIs of proven effectiveness need to be implemented on a much broader scale. Even if one single intervention helps selected patients only, adopting more such solutions in daily care is definitely worth trying. In other words, in lack of a magic wand that could eliminate non-adherence, we need to make the most of available innovations.
